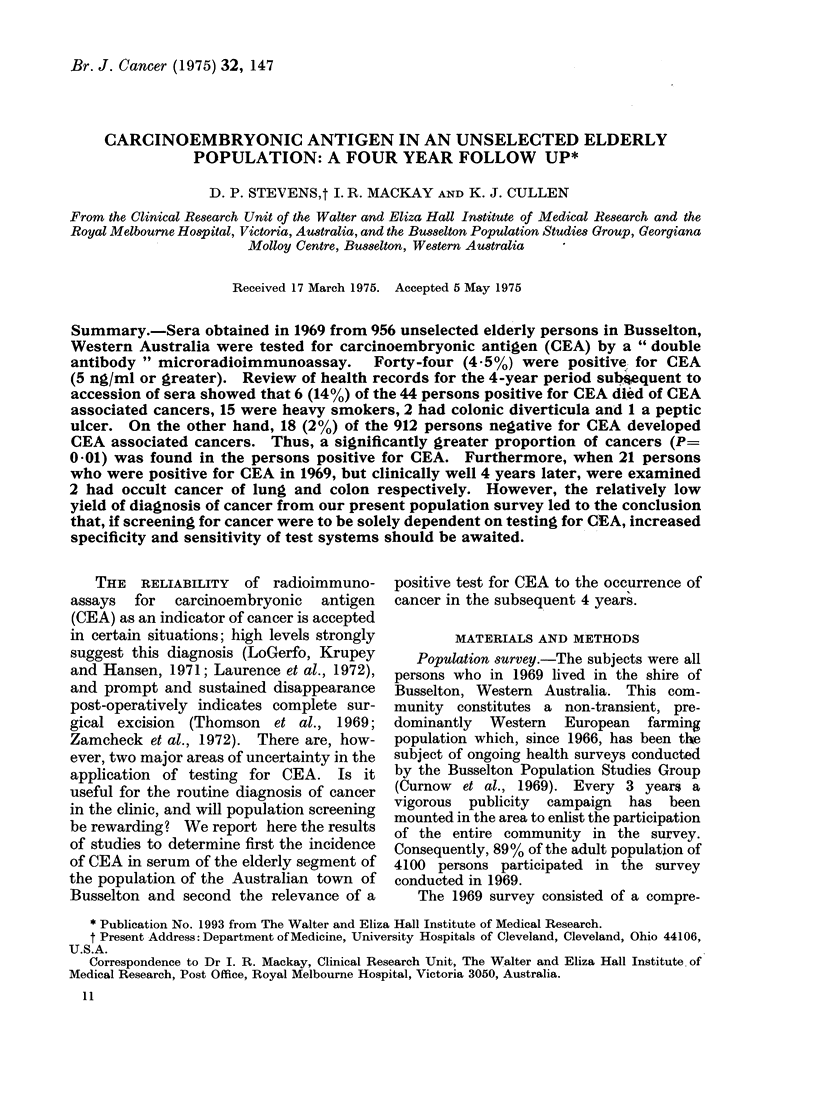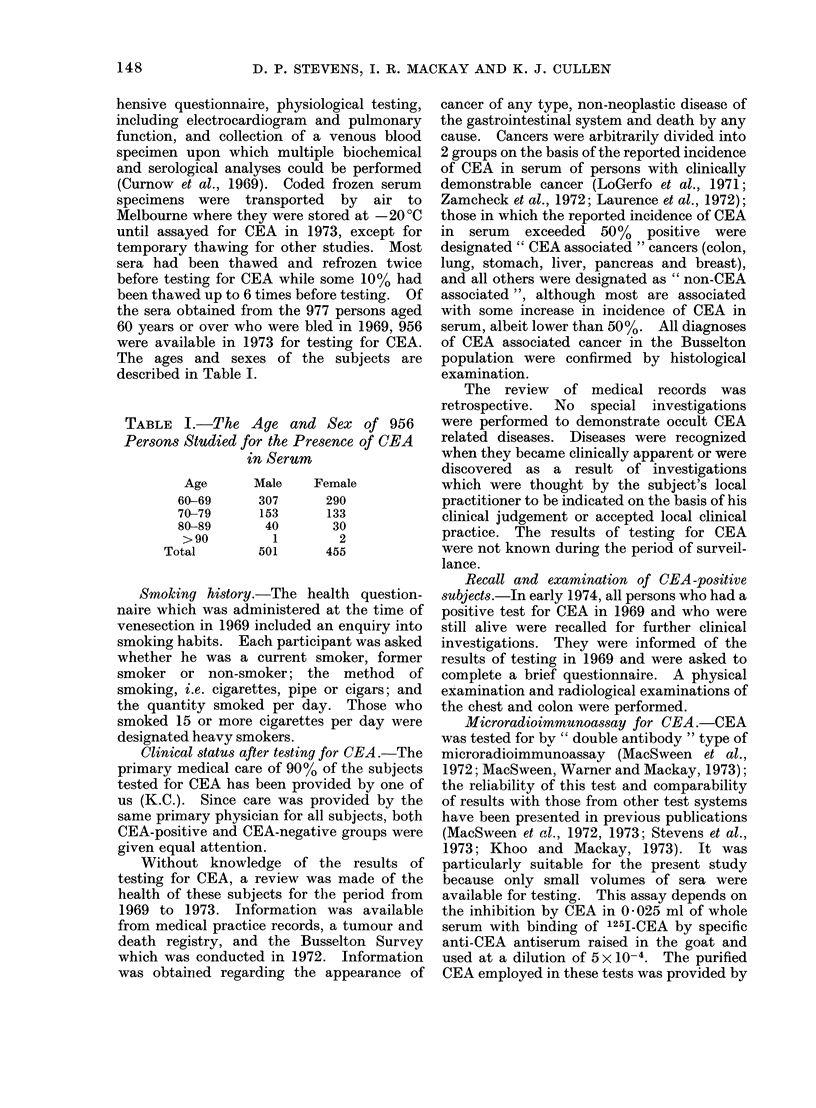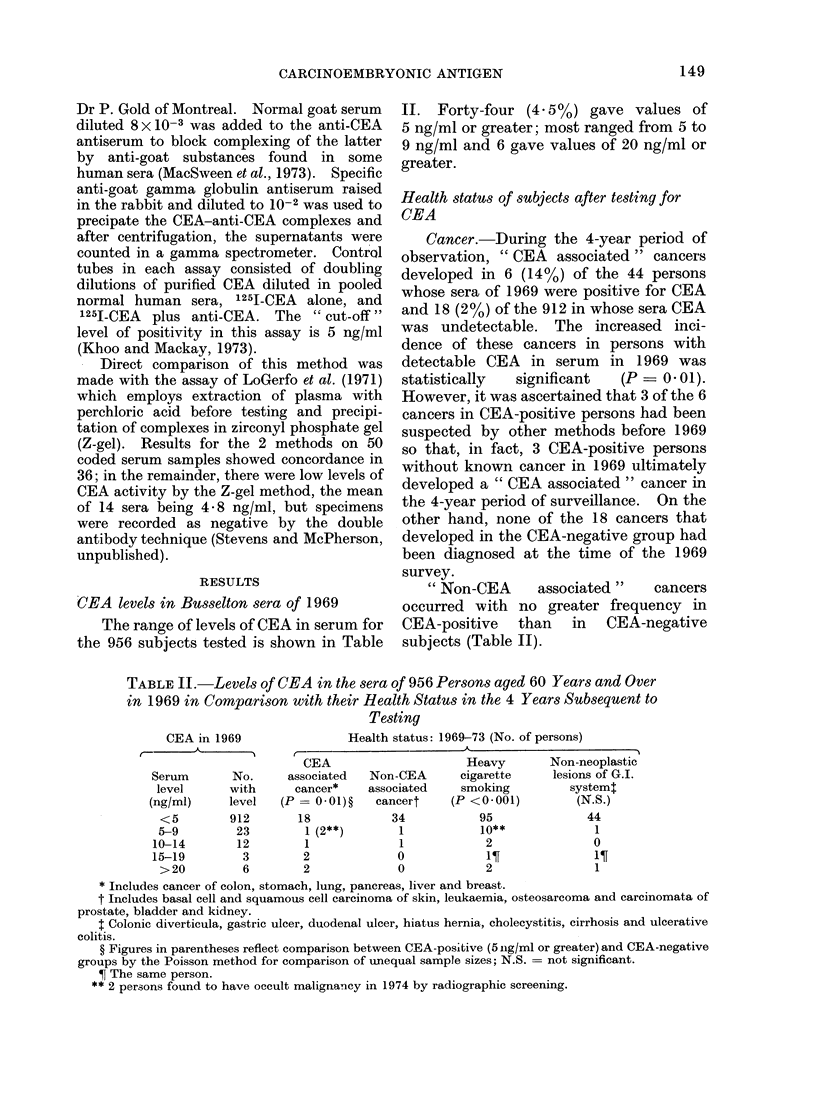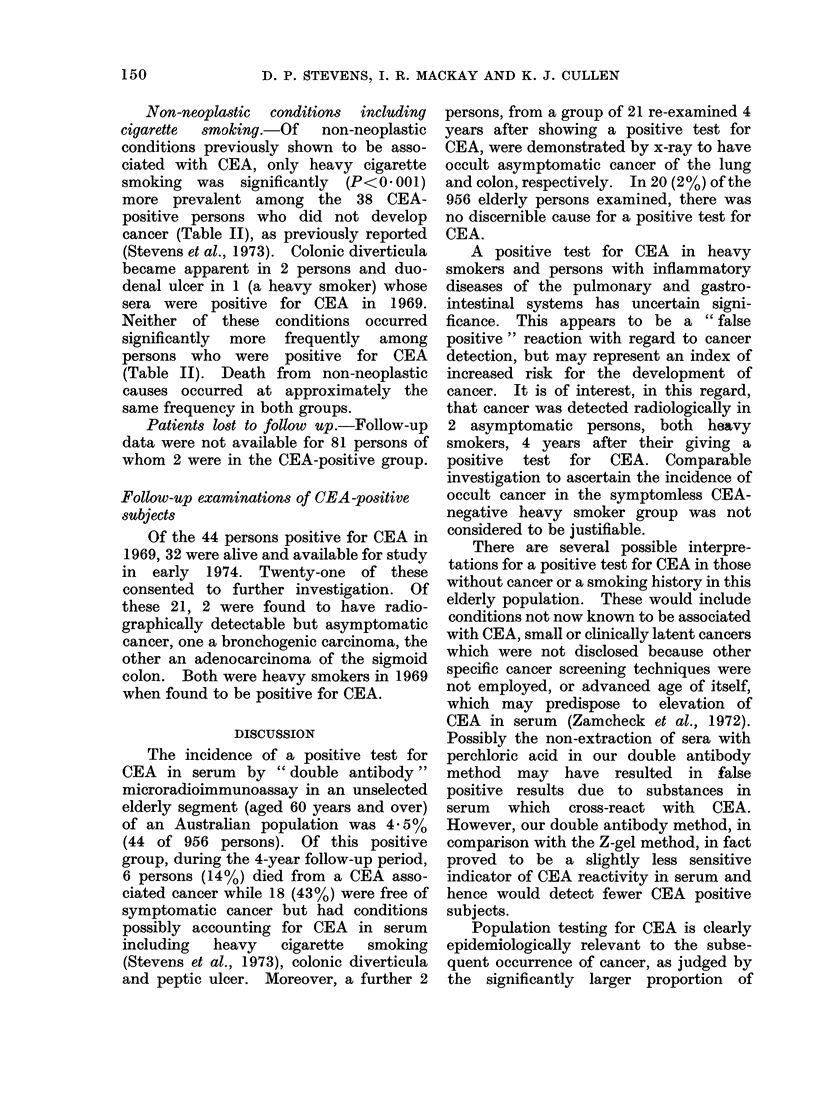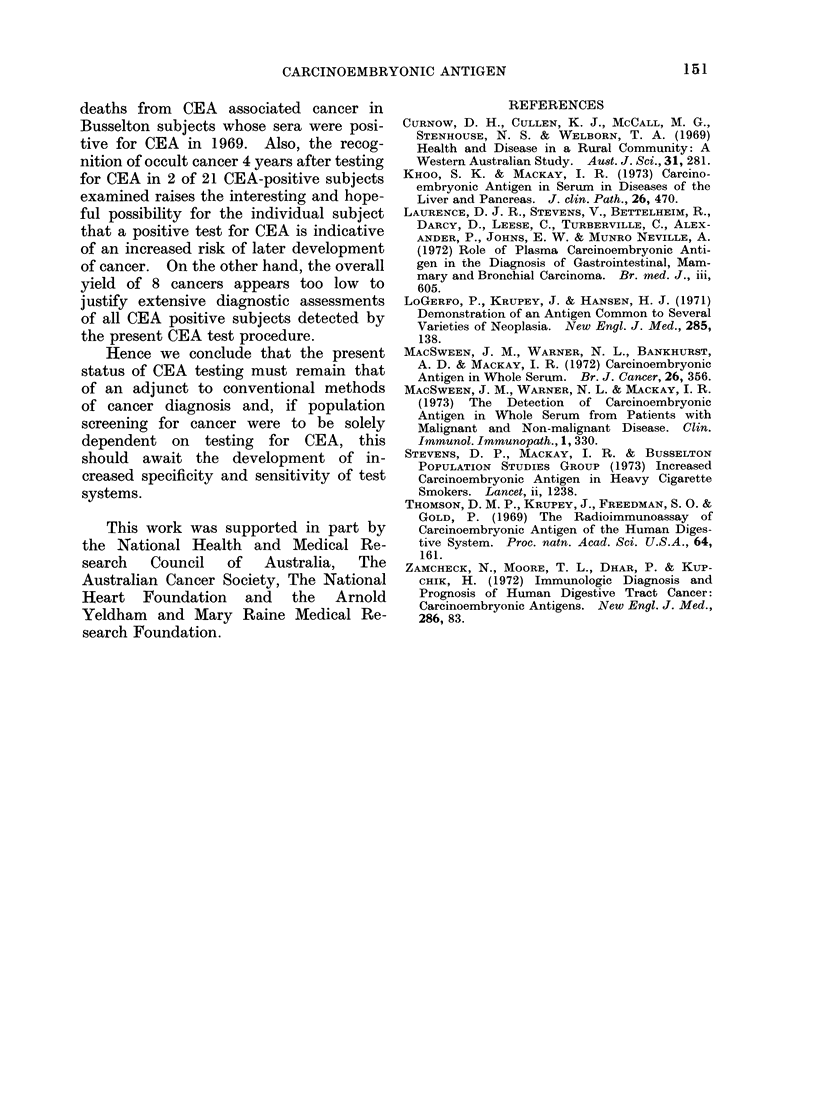# Carcinoembryonic antigen in an unselected elderly population: a four year follow up.

**DOI:** 10.1038/bjc.1975.143

**Published:** 1975-08

**Authors:** D. P. Stevens, I. R. Mackay, K. J. Cullen

## Abstract

Sera obtained in 1969 from 956 unselected elderly persons in Busselton, Western Australia were tested for carcinoembryonic antigen (CEA) by a "double antibody" microradioimmunoassay. Forty-four (4-5%) were positive for CEA (5 ng/ml or greater). Review of health records for the 4-year period subsequent to accession of sera showed that 6 (14%) of the 44 persons positive for CEA died of CEA associated cancers, 15 were heavy smokers, 2 had colonic diverticula and 1 a peptic ulcer. On the other hand, 18 (2%) of the 912 persons negative for CEA developed CEA associated cancers. Thus, a significantly greater proportion of cancers (P = 0-01) was found in the persons positive for CEA. Furthermore, when 21 persons who were positive for CEA in 1969, but clinically well 4 years later, were examined 2 had occult cancer of lung and colon respectively. However, the relatively low yield of diagnosis of cancer from our present population survey led to the conclusion that, if screening for cancer were to be solely dependent on testing for CEA, increased specificity and sensitivity of test systems should be awaited.


*Br. J. Cancer* (1975) **32,** 147

### CARCINOEMBRYONIC ANTIGEN IN AN UNSELECTED ELDERLY POPULATION: A FOUR YEAR FOLLOW UP*

D. P. STEVENS,† I. R. MACKAY AND K. J. CULLEN

*From the Clinical Research Unit of the Walter and Eliza Hall Institute of Medical Research and the Royal Melbourne Hospital, Victoria, Australia, and the Busselton Population Studies Group, Georgiana Molloy Centre, Busselton, Western Australia*

Received 17 March 1975. Accepted 5 May 1975

**Summary.—Sera obtained in 1969 from 956 unselected elderly persons in Busselton, Western Australia were tested for carcinoembryonic antigen (CEA) by a "double antibody" microradioimmunoassay. Forty-four (4·5%) were positive for CEA (5 ng/ml or greater). Review of health records for the 4-year period subsequent to accession of sera showed that 6 (14%) of the 44 persons positive for CEA died of CEA associated cancers, 15 were heavy smokers, 2 had colonic diverticula and 1 a peptic ulcer. On the other hand, 18 (2%) of the 912 persons negative for CEA developed CEA associated cancers. Thus, a significantly greater proportion of cancers ($P=$ 0·01) was found in the persons positive for CEA. Furthermore, when 21 persons who were positive for CEA in 1969, but clinically well 4 years later, were examined 2 had occult cancer of lung and colon respectively. However, the relatively low yield of diagnosis of cancer from our present population survey led to the conclusion that, if screening for cancer were to be solely dependent on testing for CEA, increased specificity and sensitivity of test systems should be awaited.**

THE RELIABILITY of radioimmunoassays for carcinoembryonic antigen (CEA) as an indicator of cancer is accepted in certain situations; high levels strongly suggest this diagnosis (LoGerfo, Krupey and Hansen, 1971; Laurence *et al.*, 1972), and prompt and sustained disappearance post-operatively indicates complete surgical excision (Thomson *et al.*, 1969; Zamcheck *et al.*, 1972). There are, however, two major areas of uncertainty in the application of testing for CEA. Is it useful for the routine diagnosis of cancer in the clinic, and will population screening be rewarding? We report here the results of studies to determine first the incidence of CEA in serum of the elderly segment of the population of the Australian town of Busselton and second the relevance of a positive test for CEA to the occurrence of cancer in the subsequent 4 years.

### MATERIALS AND METHODS

*Population survey.*—The subjects were all persons who in 1969 lived in the shire of Busselton, Western Australia. This community constitutes a non-transient, predominantly Western European farming population which, since 1966, has been the subject of ongoing health surveys conducted by the Busselton Population Studies Group (Curnow *et al.*, 1969). Every 3 years a vigorous publicity campaign has been mounted in the area to enlist the participation of the entire community in the survey. Consequently, 89% of the adult population of 4100 persons participated in the survey conducted in 1969.

The 1969 survey consisted of a compre-

* Publication No. 1993 from The Walter and Eliza Hall Institute of Medical Research.

† Present Address: Department of Medicine, University Hospitals of Cleveland, Cleveland, Ohio 44106, U.S.A.

Correspondence to Dr I. R. Mackay, Clinical Research Unit, The Walter and Eliza Hall Institute of Medical Research, Post Office, Royal Melbourne Hospital, Victoria 3050, Australia.

hensive questionnaire, physiological testing, including electrocardiogram and pulmonary function, and collection of a venous blood specimen upon which multiple biochemical and serological analyses could be performed (Curnow *et al.*, 1969). Coded frozen serum specimens were transported by air to Melbourne where they were stored at −20 °C until assayed for CEA in 1973, except for temporary thawing for other studies. Most sera had been thawed and refrozen twice before testing for CEA while some 10% had been thawed up to 6 times before testing. Of the sera obtained from the 977 persons aged 60 years or over who were bled in 1969, 956 were available in 1973 for testing for CEA. The ages and sexes of the subjects are described in Table I.

TABLE I.—*The Age and Sex of* 956 *Persons Studied for the Presence of CEA in Serum*

| Age | Male | Female |
|---|---|---|
| 60–69 | 307 | 290 |
| 70–79 | 153 | 133 |
| 80–89 | 40 | 30 |
| >90 | 1 | 2 |
| Total | 501 | 455 |

*Smoking history.*—The health questionnaire which was administered at the time of venesection in 1969 included an enquiry into smoking habits. Each participant was asked whether he was a current smoker, former smoker or non-smoker; the method of smoking, *i.e.* cigarettes, pipe or cigars; and the quantity smoked per day. Those who smoked 15 or more cigarettes per day were designated heavy smokers.

*Clinical status after testing for CEA.*—The primary medical care of 90% of the subjects tested for CEA has been provided by one of us (K.C.). Since care was provided by the same primary physician for all subjects, both CEA-positive and CEA-negative groups were given equal attention.

Without knowledge of the results of testing for CEA, a review was made of the health of these subjects for the period from 1969 to 1973. Information was available from medical practice records, a tumour and death registry, and the Busselton Survey which was conducted in 1972. Information was obtained regarding the appearance of cancer of any type, non-neoplastic disease of the gastrointestinal system and death by any cause. Cancers were arbitrarily divided into 2 groups on the basis of the reported incidence of CEA in serum of persons with clinically demonstrable cancer (LoGerfo *et al.*, 1971; Zamcheck *et al.*, 1972; Laurence *et al.*, 1972); those in which the reported incidence of CEA in serum exceeded 50% positive were designated " CEA associated " cancers (colon, lung, stomach, liver, pancreas and breast), and all others were designated as " non-CEA associated ", although most are associated with some increase in incidence of CEA in serum, albeit lower than 50%. All diagnoses of CEA associated cancer in the Busselton population were confirmed by histological examination.

The review of medical records was retrospective. No special investigations were performed to demonstrate occult CEA related diseases. Diseases were recognized when they became clinically apparent or were discovered as a result of investigations which were thought by the subject's local practitioner to be indicated on the basis of his clinical judgement or accepted local clinical practice. The results of testing for CEA were not known during the period of surveillance.

*Recall and examination of CEA-positive subjects.*—In early 1974, all persons who had a positive test for CEA in 1969 and who were still alive were recalled for further clinical investigations. They were informed of the results of testing in 1969 and were asked to complete a brief questionnaire. A physical examination and radiological examinations of the chest and colon were performed.

*Microradioimmunoassay for CEA.*—CEA was tested for by " double antibody " type of microradioimmunoassay (MacSween *et al.*, 1972; MacSween, Warner and Mackay, 1973); the reliability of this test and comparability of results with those from other test systems have been presented in previous publications (MacSween *et al.*, 1972, 1973; Stevens *et al.*, 1973; Khoo and Mackay, 1973). It was particularly suitable for the present study because only small volumes of sera were available for testing. This assay depends on the inhibition by CEA in 0·025 ml of whole serum with binding of $^{125}$I-CEA by specific anti-CEA antiserum raised in the goat and used at a dilution of $5 \times 10^{-4}$. The purified CEA employed in these tests was provided by

Dr P. Gold of Montreal. Normal goat serum diluted $8 \times 10^{-3}$ was added to the anti-CEA antiserum to block complexing of the latter by anti-goat substances found in some human sera (MacSween *et al.*, 1973). Specific anti-goat gamma globulin antiserum raised in the rabbit and diluted to $10^{-2}$ was used to precipate the CEA–anti-CEA complexes and after centrifugation, the supernatants were counted in a gamma spectrometer. Control tubes in each assay consisted of doubling dilutions of purified CEA diluted in pooled normal human sera, $^{125}$I-CEA alone, and $^{125}$I-CEA plus anti-CEA. The " cut-off " level of positivity in this assay is 5 ng/ml (Khoo and Mackay, 1973).

Direct comparison of this method was made with the assay of LoGerfo *et al.* (1971) which employs extraction of plasma with perchloric acid before testing and precipitation of complexes in zirconyl phosphate gel (Z-gel). Results for the 2 methods on 50 coded serum samples showed concordance in 36; in the remainder, there were low levels of CEA activity by the Z-gel method, the mean of 14 sera being 4·8 ng/ml, but specimens were recorded as negative by the double antibody technique (Stevens and McPherson, unpublished).

### RESULTS

### *CEA levels in Busselton sera of* 1969

The range of levels of CEA in serum for the 956 subjects tested is shown in Table II. Forty-four (4·5%) gave values of 5 ng/ml or greater; most ranged from 5 to 9 ng/ml and 6 gave values of 20 ng/ml or greater.

### *Health status of subjects after testing for CEA*

*Cancer.*—During the 4-year period of observation, " CEA associated " cancers developed in 6 (14%) of the 44 persons whose sera of 1969 were positive for CEA and 18 (2%) of the 912 in whose sera CEA was undetectable. The increased incidence of these cancers in persons with detectable CEA in serum in 1969 was statistically significant ($P = 0 \cdot 01$). However, it was ascertained that 3 of the 6 cancers in CEA-positive persons had been suspected by other methods before 1969 so that, in fact, 3 CEA-positive persons without known cancer in 1969 ultimately developed a " CEA associated " cancer in the 4-year period of surveillance. On the other hand, none of the 18 cancers that developed in the CEA-negative group had been diagnosed at the time of the 1969 survey.

" Non-CEA associated " cancers occurred with no greater frequency in CEA-positive than in CEA-negative subjects (Table II).

TABLE II.—*Levels of CEA in the sera of* 956 *Persons aged* 60 *Years and Over in* 1969 *in Comparison with their Health Status in the* 4 *Years Subsequent to Testing*

| CEA in 1969 | | Health status: 1969–73 (No. of persons) | | | |
|---|---|---|---|---|---|
| Serum level (ng/ml) | No. with level | CEA associated cancer* ($P = 0 \cdot 01$)§ | Non-CEA associated cancer† | Heavy cigarette smoking ($P < 0 \cdot 001$) | Non-neoplastic lesions of G.I. system‡ (N.S.) |
| <5 | 912 | 18 | 34 | 95 | 44 |
| 5–9 | 23 | 1 (2**) | 1 | 10** | 1 |
| 10–14 | 12 | 1 | 1 | 2 | 0 |
| 15–19 | 3 | 2 | 0 | 1¶ | 1¶ |
| >20 | 6 | 2 | 0 | 2 | 1 |

* Includes cancer of colon, stomach, lung, pancreas, liver and breast.

† Includes basal cell and squamous cell carcinoma of skin, leukaemia, osteosarcoma and carcinomata of prostate, bladder and kidney.

‡ Colonic diverticula, gastric ulcer, duodenal ulcer, hiatus hernia, cholecystitis, cirrhosis and ulcerative colitis.

§ Figures in parentheses reflect comparison between CEA-positive (5 ng/ml or greater) and CEA-negative groups by the Poisson method for comparison of unequal sample sizes; N.S. = not significant.

¶ The same person.

** 2 persons found to have occult malignancy in 1974 by radiographic screening.

*Non-neoplastic conditions including cigarette smoking.*—Of non-neoplastic conditions previously shown to be associated with CEA, only heavy cigarette smoking was significantly ($P<0 \cdot 001$) more prevalent among the 38 CEA-positive persons who did not develop cancer (Table II), as previously reported (Stevens *et al.*, 1973). Colonic diverticula became apparent in 2 persons and duodenal ulcer in 1 (a heavy smoker) whose sera were positive for CEA in 1969. Neither of these conditions occurred significantly more frequently among persons who were positive for CEA (Table II). Death from non-neoplastic causes occurred at approximately the same frequency in both groups.

*Patients lost to follow up.*—Follow-up data were not available for 81 persons of whom 2 were in the CEA-positive group.

*Follow-up examinations of CEA-positive subjects*

Of the 44 persons positive for CEA in 1969, 32 were alive and available for study in early 1974. Twenty-one of these consented to further investigation. Of these 21, 2 were found to have radiographically detectable but asymptomatic cancer, one a bronchogenic carcinoma, the other an adenocarcinoma of the sigmoid colon. Both were heavy smokers in 1969 when found to be positive for CEA.

### DISCUSSION

The incidence of a positive test for CEA in serum by " double antibody " microradioimmunoassay in an unselected elderly segment (aged 60 years and over) of an Australian population was $4 \cdot 5\%$ (44 of 956 persons). Of this positive group, during the 4-year follow-up period, 6 persons (14%) died from a CEA associated cancer while 18 (43%) were free of symptomatic cancer but had conditions possibly accounting for CEA in serum including heavy cigarette smoking (Stevens *et al.*, 1973), colonic diverticula and peptic ulcer. Moreover, a further 2 persons, from a group of 21 re-examined 4 years after showing a positive test for CEA, were demonstrated by x-ray to have occult asymptomatic cancer of the lung and colon, respectively. In 20 (2%) of the 956 elderly persons examined, there was no discernible cause for a positive test for CEA.

A positive test for CEA in heavy smokers and persons with inflammatory diseases of the pulmonary and gastrointestinal systems has uncertain significance. This appears to be a " false positive " reaction with regard to cancer detection, but may represent an index of increased risk for the development of cancer. It is of interest, in this regard, that cancer was detected radiologically in 2 asymptomatic persons, both heavy smokers, 4 years after their giving a positive test for CEA. Comparable investigation to ascertain the incidence of occult cancer in the symptomless CEA-negative heavy smoker group was not considered to be justifiable.

There are several possible interpretations for a positive test for CEA in those without cancer or a smoking history in this elderly population. These would include conditions not now known to be associated with CEA, small or clinically latent cancers which were not disclosed because other specific cancer screening techniques were not employed, or advanced age of itself, which may predispose to elevation of CEA in serum (Zamcheck *et al.*, 1972). Possibly the non-extraction of sera with perchloric acid in our double antibody method may have resulted in false positive results due to substances in serum which cross-react with CEA. However, our double antibody method, in comparison with the Z-gel method, in fact proved to be a slightly less sensitive indicator of CEA reactivity in serum and hence would detect fewer CEA positive subjects.

Population testing for CEA is clearly epidemiologically relevant to the subsequent occurrence of cancer, as judged by the significantly larger proportion of deaths from CEA associated cancer in Busselton subjects whose sera were positive for CEA in 1969. Also, the recognition of occult cancer 4 years after testing for CEA in 2 of 21 CEA-positive subjects examined raises the interesting and hopeful possibility for the individual subject that a positive test for CEA is indicative of an increased risk of later development of cancer. On the other hand, the overall yield of 8 cancers appears too low to justify extensive diagnostic assessments of all CEA positive subjects detected by the present CEA test procedure.

Hence we conclude that the present status of CEA testing must remain that of an adjunct to conventional methods of cancer diagnosis and, if population screening for cancer were to be solely dependent on testing for CEA, this should await the development of increased specificity and sensitivity of test systems.

This work was supported in part by the National Health and Medical Research Council of Australia, The Australian Cancer Society, The National Heart Foundation and the Arnold Yeldham and Mary Raine Medical Research Foundation.

REFERENCES

CURNOW, D. H., CULLEN, K. J., McCALL, M. G., STENHOUSE, N. S. & WELBORN, T. A. (1969) Health and Disease in a Rural Community: A Western Australian Study. *Aust. J. Sci.*, **31**, 281.

KHOO, S. K. & MACKAY, I. R. (1973) Carcinoembryonic Antigen in Serum in Diseases of the Liver and Pancreas. *J. clin. Path.*, **26**, 470.

LAURENCE, D. J. R., STEVENS, V., BETTELHEIM, R., DARCY, D., LEESE, C., TURBERVILLE, C., ALEXANDER, P., JOHNS, E. W. & MUNRO NEVILLE, A. (1972) Role of Plasma Carcinoembryonic Antigen in the Diagnosis of Gastrointestinal, Mammary and Bronchial Carcinoma. *Br. med. J.*, iii, 605.

LOGERFO, P., KRUPEY, J. & HANSEN, H. J. (1971) Demonstration of an Antigen Common to Several Varieties of Neoplasia. *New Engl. J. Med.*, **285**, 138.

MACSWEEN, J. M., WARNER, N. L., BANKHURST, A. D. & MACKAY, I. R. (1972) Carcinoembryonic Antigen in Whole Serum. *Br. J. Cancer*, **26**, 356.

MACSWEEN, J. M., WARNER, N. L. & MACKAY, I. R. (1973) The Detection of Carcinoembryonic Antigen in Whole Serum from Patients with Malignant and Non-malignant Disease. *Clin. Immunol. Immunopath.*, **1**, 330.

STEVENS, D. P., MACKAY, I. R. & BUSSELTON POPULATION STUDIES GROUP (1973) Increased Carcinoembryonic Antigen in Heavy Cigarette Smokers. *Lancet*, ii, 1238.

THOMSON, D. M. P., KRUPEY, J., FREEDMAN, S. O. & GOLD, P. (1969) The Radioimmunoassay of Carcinoembryonic Antigen of the Human Digestive System. *Proc. natn. Acad. Sci. U.S.A.*, **64**, 161.

ZAMCHECK, N., MOORE, T. L., DHAR, P. & KUPCHIK, H. (1972) Immunologic Diagnosis and Prognosis of Human Digestive Tract Cancer: Carcinoembryonic Antigens. *New Engl. J. Med.*, **286**, 83.